# Myxedema Coma: A Grave Phenomenon Partially Reversed CKD Status With Treatment of Hypothyroidism

**DOI:** 10.7759/cureus.40221

**Published:** 2023-06-10

**Authors:** Saikiran Mandyam, Sai Sudha Valisekka, Devam Parghi, Yagnapriya Chirrareddy, Pavan Kumar Reddy Kalluru, Nowoghomwenma C Ibie

**Affiliations:** 1 Internal Medicine, Southeast Health Medical Center, Dothan, USA; 2 Internal Medicine, University of Minnesota, Minneapolis, USA; 3 Internal Medicine, Sri Padmavathi Medical College, Tirupati, IND; 4 Internal Medicine, Sri Venakteswara Medical college, Tirupati, IND; 5 Nephrology, Southeast Health Medical Center, Dothan, USA

**Keywords:** tsh, t4, t3, improved ckd stage, hypothyroid myxedema coma

## Abstract

Myxedema coma is a grave medical condition that warrants emergent medical management to avoid adverse effects and unfavorable outcomes. Intravenous thyroid hormones (T3 and T4), along with intravenous hydrocortisone and frequent vital monitoring, are the mainstays of the management of myxedema coma. The interplay between CKD and hypothyroidism is fascinating and can affect each other. It is often very difficult for physicians to differentiate between sepsis and myxedema coma, especially in the early stages. Infections and medication non-compliance are the leading causes of precipitation myxedema coma. We describe a case report presented with myxedema coma and CKD, which was successfully managed and also led to a partial reversal of CKD status.

## Introduction

Myxedema coma is a grave medical condition and a medical emergency that is defined as severe hypothyroidism leading to slowing of function in multiple organs [1]. This medical condition needs urgent medical management as mortality is high [2]. Elderly women are most frequently diagnosed with myxedema coma, although the name "coma" does not necessarily mean patients are comatosed; the usual neurological manifestations involve lethargy and confusion [3,4].

In the literature, predialysis patients with chronic kidney diseases are associated with hypothyroidism, mostly subclinical [5,6]. Kidneys play a major role in the excretion of iodide, which in turn accumulates in CKD patients and contributes to hypothyroidism [7]. There was some evidence of reversible CKD status with appropriate management of hypothyroidism [8]. Hypothyroidism itself rarely presents as a myxedema coma. Through this case report, we demonstrate a myxedema coma case that was successfully managed with intravenous T3 and T4 and partially reversed CKD status with the management of underlying hypothyroidism.

## Case presentation

**Figure 1:**
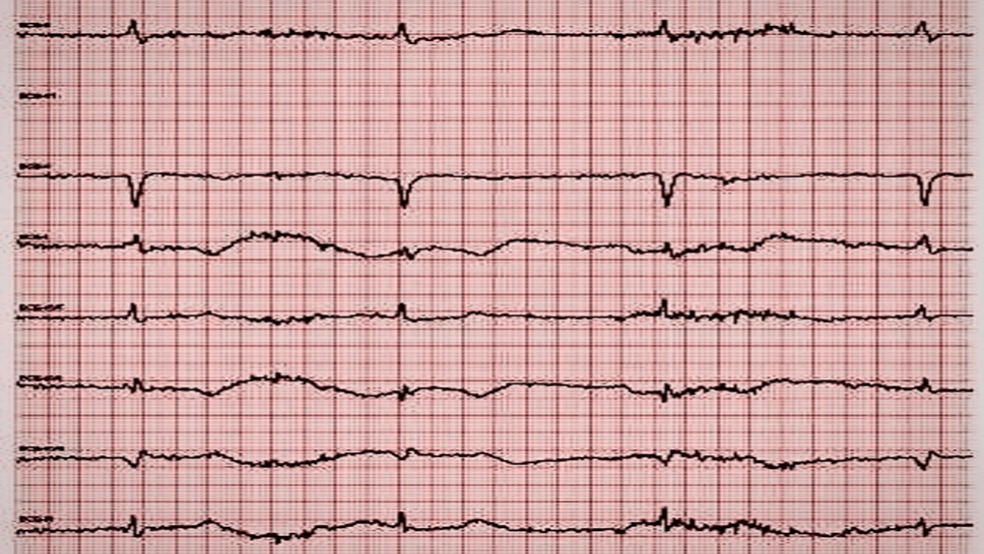
A telemetry strip demonstrating sinus bradycardia with a heart rate of 38

**Figure 2:**
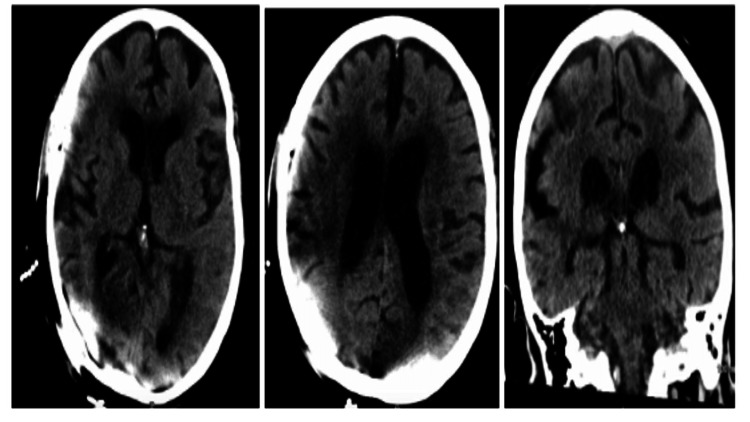
CT head without contrast showed no evidence of an acute abnormality contributing to the patient’s encephalopathy

**Figure 3:**
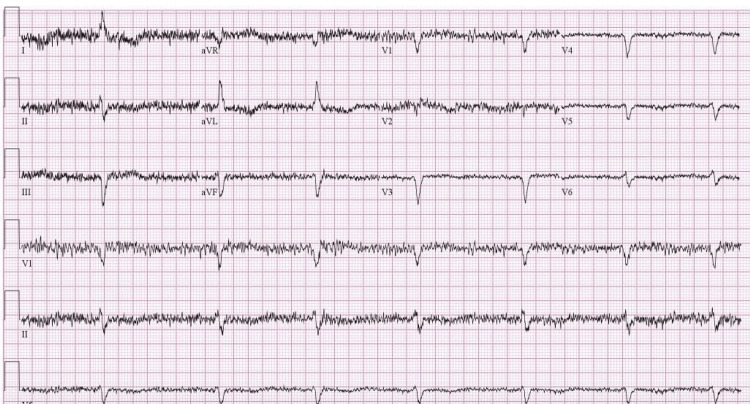
EKG before discharge demonstrating regular QRS complexes, presumed sinus rhythm due to artifact as telemetry strips showed p-waves QRS: electrocardiographic complex consisting of the Q, R, and S waves.

A 79-year-old female with a past medical history of congestive heart failure (grade 2 diastolic dysfunction with preserved left ventricular ejection fraction), atrial fibrillation on anticoagulation, and chronic kidney disease stage 5 presented to our hospital due to worsening confusion and decreased appetite. Upon questioning the family (the patient was altered), the patient was confused and disoriented for the past four days before hospitalization. The patient’s vitals were significant for bradycardia (35-40) and borderline low blood pressure. Her physical examination upon arrival at our hospital demonstrated normal heart sounds, no gross focal neurological deficits, no jugular venous distension, and bilateral normal breath sounds with no increased work of breathing. Initial telemetry strips (Figure 1) showed significant sinus bradycardia with non-specific intraventricular delay. Blood work showed a white count that was within normal limits. The urine toxicology screen was negative. Initial thyroid stimulating hormone (TSH) was very high, and T3 and T4 were undetectable (Table 1). Cortisol AM was within normal limits. A CT head without IV contrast (Figure 2) showed no acute abnormality. Arterial blood gas analysis (ABG) showed normal PH and normal PCO2 levels. Vitamin B12, folate, vitamin D levels, ammonia, liver function tests, and blood glucose levels were within normal limits. Urinalysis, chest X-ray, and electrolytes were unremarkable. The patient met the criteria for myxedema coma, was transferred to the intensive care unit for close monitoring, and started hydrocortisone 100 mg every eight hours. Intravenous T4 (200 mcg bolus followed by 50 mcg daily) and T3 (5 mcg bolus followed by 2.5 mcg every eight hours) were also started. TSH improved over the hospital course (Table 1). The patient was initially started on dopamine infusion for bradycardia and hypotension, along with intravenous thyroid. Dopamine was successfully weaned off within the first six hours, and the heart rate remained above 60 beats per minute. There was a significant change in her mental status; the patient was more talkative, alert, and oriented x3 within the first 48 hours of the presentation. Hydrocortisone was tapered to prednisone oral (50 mg for three days followed by 20 mg for three days) and then discontinued. T4 was continued at a dose of 75 mcg orally. T3 was discontinued. Later on, after her mental status improved, the patient reported she had not been taking her oral medications (including levothyroxine 75 micrograms orally once daily) for approximately six months, which is likely the cause of her precipitating myxedema coma. The patient also reported that she discussed getting initiated on dialysis, which she denied in the past as well. Her home levothyroxine has been scheduled. Interestingly, serum creatinine has significantly improved, and at the time of discharge, her creatinine was 1.51, with an estimated GFR of 35 ml/min/1.73 m2. She was admitted to the hospital with acute kidney injury (AKI) on chronic kidney disease (CKD) stage 5, and FeNa was suggestive of prerenal AKI. The patient received judicious fluids given her heart failure history, although the degree of correction of her creatinine does not justify the dehydration and prerenal AKI components alone. We think a component of severe hypothyroidism played a major role in her underlying progression of CKD, which was partially reversed with appropriate management of hypothyroidism. Patient’s prior labs (Table 1) demonstrated CKD stages 4 and 5 approximately two years and five months before this hospitalization, respectively. An EKG (Figure 3) was repeated before discharge and showed normal sinus rhythm with non-specific intraventricular delay. The patient was safely discharged to a rehab facility with recommendations to repeat thyroid labs and kidney function tests within one week after discharge on levothyroxine 75 micrograms orally daily.

**Table 1:** Tabular format of labs through the hospital course 1. TSH (mIU/L): Thyroid stimulating hormone in milli international units per liter; 2. T3 (ng/dl): Triiodothyronine in nanogram per deciliter, 3. T4(ng/dl): Thyroxine; 4. Cr (mg/dl): Creatinine in milligram per deciliter; 5. GFR (ml/min/1.73m2): Glomerular filtration rate in milliliter per min per 1.73 square meters.

Lab/ Day of admission	2 years prior	5 months prior	0	1	2	3	4	5	6	7	8	9	10
TSH (mIU/L)	-	-	331	-	39.3	-	-	19.4	-	-	-	-	-
T3 (ng/dl)	-	-	-	-	18.3	-	-	2.47	-	-	-	-	-
T4 (ng/dl)	-	-	less than 0.25	-	0.78	-	-	0.66	-	-	-	-	-
Creatinine (mg/dl)	3.7 ( classified as CKD 4)	4.52 ( classified as CKD 5)	6.78	6.16	5.49	4.78	3.66	2.73	2.07	2.03	1.64	1.66	1.51
Gfr ml/min/1.73 m^2^	16	7.6	5.8	6.5	7.4	8.8	12.1	17.2	24	24.6	31.7	31.3	35

## Discussion

Myxedema coma can present as a progression of untreated hypothyroidism or can be precipitated by infections, burns, trauma, hypoglycemia, hypothermia, medications like amiodarone, lithium, and anesthetics [9]. It is a medical emergency that is defined as severe hypothyroidism leading to a slowing of function in multiple organs. It includes a wide range of clinical manifestations that include neurological, cardiovascular, respiratory, endocrine, and electrolyte derangements [1]. Myxedema coma is suspected when patients present with decreased mentation, hypothermia and hyponatremia, along with additional symptoms of hypothyroidism. Management and diagnostic workup are well published in the literature for myxedema coma. In this article, we focus on the interplay between chronic kidney disease and hypothyroidism. 

Chronic kidney disease (CKD) is a highly debilitating condition that significantly impacts the well-being of more than 37 million adults in the United States. The economic burden associated with CKD is substantial, with estimated annual Medicare costs reaching up to US$84 billion [10]. A comprehensive study by the National Health and Nutrition Examination Survey (NHANES III), which encompasses a representative sample of the US population, revealed that a considerable proportion of CKD patients, ranging from 11% to 23%, also experience hypothyroidism [5]. 

CKD has a significant impact on the hypothalamic-pituitary-thyroid axis. It can contribute to thyroid dysfunction through various mechanisms. Uremia, a condition characterized by the buildup of urea and other waste products in the blood, can lead to a decrease in the production of thyrotropin-releasing hormone (TRH), which is responsible for stimulating the release of thyroid-stimulating hormone (TSH) [11]. Metabolic acidosis, another common issue in renal dysfunction, can cause alterations in thyroid function, resulting in elevated levels of TSH and reduced levels of thyroxine (T4) and/or triiodothyronine (T3) [12]. Additionally, impaired renal function can lead to reduced clearance of iodine, resulting in iodine retention in the body [13]. One very well-established mechanism in patients with CKD is the reduction in glomerular filtration rate (GFR), which leads to a decrease in the clearance of inflammatory cytokines such as TNF-alpha and IL-1. As a consequence, the expression of type-1 5'-deiodinase is inhibited, further diminishing the conversion of thyroxine (T4) to the active form, triiodothyronine (T3). This ultimately results in low levels of T3, which is a common finding in CKD. Additionally, the decreased GFR can impede the clearance of iodine and certain goitrogenic substances, contributing to the development of hypothyroidism and myxedema coma. However, further research is needed to investigate the potential causal mechanisms underlying the association between chronic kidney disease (CKD) and elevated TSH levels, along with decreased thyroid function. This includes exploring the potential involvement of factors such as autoimmunity and excessive iodine intake.

On the other hand, there have been numerous reports suggesting that kidney function can be affected by thyroid disorders (Figure 4), both in cases of hyperthyroidism and hypothyroidism. Treatment for hypothyroidism has been associated with an increase in the estimated glomerular filtration rate (eGFR) [14], while treatment for hyperthyroidism has been linked to a decrease in eGFR. Several mechanisms have been proposed to explain these effects, including changes in renal perfusion pressure, the renin-angiotensin-aldosterone system, tubular ion transporters, the tubuloglomerular feedback system, and creatinine metabolism [7-10]. These mechanisms involve both pathways dependent on glomerular filtration rate (GFR) and pathways independent of GFR. Studies have shown that GFR actually changes before and after treatment for thyroid dysfunction [15]. This indicates a correlation between changes in GFR and thyroid disorders. Consequently, the treatment for thyroid dysfunction using levothyroxine may also impact the status of chronic kidney disease (CKD) [16].

Treatment of hypothyroidism can reverse CKD in a significant portion of cases, with 35% transitioning from CKD to non-CKD status. Properly managing thyroid dysfunction can improve renal function, particularly in cases of CKD with hypothyroidism [15]. It is crucial to include hypothyroidism evaluation in routine CKD analyses due to its potential to slow down CKD progression. However, treating patients with mildly elevated TSH levels (below 20 IU/mL) can lead to muscle breakdown and a negative nitrogen balance. In addition to thyroid hormone replacement, the management of CKD in these patients should focus on addressing the underlying kidney disease and associated risk factors. This may involve optimizing blood pressure control, managing diabetes, promoting a kidney-friendly diet, and considering medication adjustments to ensure compatibility with kidney function.

Managing myxedema coma in patients with chronic kidney disease (CKD) presents significant challenges that require careful consideration. One of the primary obstacles is the altered pharmacokinetics of levothyroxine, the mainstay of treatment for hypothyroidism. CKD affects drug absorption, distribution, metabolism, and elimination, leading to variable levothyroxine bioavailability and an altered response to therapy. Consequently, close monitoring and frequent dose adjustments are crucial to achieving optimal thyroid hormone replacement and preventing under or overtreatment. Another challenge is the potential for electrolyte imbalances and fluid overload in CKD patients, which can be exacerbated during the management of myxedema coma. The delicate balance of electrolytes, particularly sodium, should be carefully maintained to avoid complications such as cerebral edema. Furthermore, fluid overload can worsen the patient's cardiovascular status. Therefore, meticulous fluid management and electrolyte monitoring are imperative. Overall, the successful management of myxedema coma in CKD patients hinges on close monitoring and timely therapy adjustment to optimize outcomes, ensuring adequate thyroid hormone replacement while minimizing the risk of electrolyte imbalances and fluid overload.

Timely diagnosis and treatment of myxedema coma in patients with CKD is of paramount importance due to the potentially life-threatening nature of this condition. Myxedema coma is a rare but severe manifestation of severe hypothyroidism that can rapidly progress and lead to irreversible organ damage if not promptly recognized and managed. CKD patients are particularly vulnerable to myxedema coma due to the interplay between thyroid dysfunction and renal impairment. Therefore, clinicians should maintain a high index of suspicion for this condition in CKD patients presenting with altered mental status, hypothermia, cardiovascular instability, and other signs of severe hypothyroidism. Prompt initiation of treatment is crucial to restoring thyroid hormone levels and metabolic function, as delayed intervention can result in devastating consequences. Timely administration of intravenous levothyroxine, along with supportive measures, including temperature regulation, fluid and electrolyte balance, and hemodynamic stability, is necessary to optimize outcomes and prevent further deterioration. The significance of early recognition and intervention cannot be overstated, as they can avert irreversible organ damage and improve the overall prognosis for patients with myxedema, coma, and CKD.

## Conclusions

Collaboration between endocrinologists, nephrologists, and other healthcare professionals is essential for the effective treatment of patients with both myxedema coma and CKD. Regular follow-up appointments and close monitoring of thyroid function and kidney parameters are necessary to ensure appropriate adjustments to medication dosages and treatment plans as the conditions evolve. In conclusion, the interplay between myxedema coma and CKD can complicate the diagnosis and treatment of affected patients. Recognizing and addressing this relationship is crucial for providing optimal care. A multidisciplinary approach tailored to thyroid hormone replacement therapy and comprehensive management of CKD is an essential component of an effective treatment strategy for individuals with both hypothyroidism and CKD.
